# A unified FLAIR hyperintensity segmentation model for various CNS tumor types and acquisition time points

**DOI:** 10.1038/s41598-026-48496-1

**Published:** 2026-04-24

**Authors:** Mathilde Gajda Faanes, David Bouget, Asgeir S. Jakola, Timothy R. Smith, Vasileios K. Kavouridis, Francesco Latini, Margret Jensdottir, Peter Milos, Henrietta Nittby Redebrandt, Rickard L. Sjöberg, Rupavathana Mahesparan, Lars Kjelsberg Pedersen, Ole Solheim, Ingerid Reinertsen

**Affiliations:** 1 Department of Health Research, SINTEF Digital, Trondheim, Norway; 2 Department of Clinical Neuroscience, Institute of Neuroscience and Physiology, University of Gothenburg, Gothenburg, Sweden; 3 Department of Neurosurgery, Sahlgrenska University Hospital, Region Västragötaland, Gothenburg, Sweden; 4 Department of Neurosurgery, Brigham and Women’s Hospital, Harvard Medical School, Boston, MA USA; 5 Department Medical Sciences, Section of Neurosurgery, Uppsala University Hospital, Uppsala, Sweden; 6 Department of Neurosurgery, Karolinska University Hospital, Stockholm, Sweden; 7 Department of Neurosurgery, Linköping University Hospital, Linköping, Sweden; 8 Department of Neurosurgery, Skåne University Hospital, Lund, Sweden; 9 Department of Clinical Science, Umeå University, Umeå, Sweden; 10 Department of Neurosurgery, Haukeland University Hospital, Bergen, Norway; 11 Department of Neurosurgery, University Hospital of North Norway, Tromsø, Norway; 12 Department of Neuromedicine and Movement Science, Norwegian University of Science and Technology, Trondheim, Norway; 13 Department of Neurosurgery, St. Olavs hospital, Trondheim University Hospital, Trondheim, Norway; 14 Department of Circulation and Medical Imaging, Norwegian University of Science and Technology, Trondheim, Norway

**Keywords:** Cancer, Computational biology and bioinformatics, Medical research, Oncology

## Abstract

Fluid-attenuated inversion recovery (FLAIR) magnetic resonance imaging (MRI) scans are important for diagnosis, treatment planning, and monitoring of various brain tumors. Depending on the tumor type, the FLAIR hyperintensity volume is an important measure to assess the tumor volume, surrounding vasogenic edema, or treatment induced changes, such as gliosis. Automatic segmentation would therefore be valuable in the clinic and in clinical trials. In this study, around 5000 FLAIR images of various brain tumors types and acquisition time points, from different neurosurgical centers, were used to train a unified FLAIR hyperintensity segmentation model using an Attention U-Net architecture. The performance was compared against dataset-specific models and was validated on different tumor types, acquisition time points, and against BraTS. The unified model achieved an average Dice score of 88.65% for pre-operative meningiomas, 80.08% for pre-operative metastases, 90.92% for pre-operative and 84.60% for post-operative gliomas from BraTS, and 84.47% for pre-operative and 61.27% for post-operative lower grade gliomas. In addition, the results showed that the unified model achieved comparable segmentation performance to the dataset-specific models on their respective datasets. The documented generalization across tumor types and acquisition time points is a strong indicator for efficient deployment in a clinical setting. The model has been integrated into Raidionics, an open-source software for CNS tumor analysis.

## Introduction

Central nervous system (CNS) tumors represent a diverse group of neoplasms and are classified by the World Health Organization (WHO) into over 130 different subtypes of brain and spinal cord tumors based on their histological and molecular characteristics^1^. The most common tumor entities are metastases, meningiomas, glioblastomas, and lower grade gliomas (IDH mutated, WHO 2–3). Magnetic resonance imaging (MRI) is a critical imaging modality for accurate diagnosis, treatment planning, evaluation of treatment responses, and longitudinal monitoring of brain tumors. Various MRI sequences are acquired to obtain information about the location, size, tumor type, and aggressiveness. T2-weighted (T2), fluid-attenuated inversion recovery (FLAIR) sequences, and T1-weighted (T1w) sequences before and after application of a gadolinium-based contrast agent (T1c), constitute the core of the standard diagnostic imaging protocol^2^. Depending on its type, the tumor may exhibit contrast enhancement, typically depicted on the T1c sequence. For contrast-enhancing tumors like metastasis, glioblastoma, and meningioma, the tumor borders and consequently the tumor volume are defined by the outer rim of T1 enhancement. For non- or less enhancing WHO grade 2–3 gliomas, the T2 or FLAIR hyperintensity (FH) volume defines the tumor borders. These tumors usually have no edema. However, for glioblastomas the FLAIR hyperintensity volume beyond the contrast-enhancing tumor has gained clinical interest. This surrounding non-enhancing FLAIR hyperintensity (SNFH) volume may represent a combination of peritumoral vasogenic edema and a gradient of infiltrating tumor cells, which can have importance for prognosis, defining the surgical target and evaluating treatment responses^3^. The current RANO Resect framework states that patients with a SNFH volume of less than 5 ml and no residual contrast enhancement exhibit the best prognoses after surgery^4^. For meningiomas, the SNFH volume beyond the T1c enhanced tumor borders is associated with WHO grade^5^, i.e malignancy, and for metastases the SNFH volume represents vasogenic edema that may cause reversible neurological deficits^6^. For any tumor undergoing radiotherapy, progressing FLAIR signals often represent treatment induced radiation gliosis. According to the new Response Assessment in Neuro-Oncology (RANO 2.0) criteria, both the contrast-enhancing and non-contrast-enhancing volumes in T1w and FLAIR MRI images are important for treatment planning and prognostication for gliomas^3^. However, delineating these volumes manually is time consuming, with high inter- and intra-observer variability^7–9^. An accurate automatic segmentation of these hyperintesities holds the potential to improve diagnostic accuracy, treatment planning, response evaluation, and prognostic evaluation, both pre- and post-operatively for various tumor types. In addition, it would be useful for imaging-based response assessment in clinical trials.

Deep learning methods are already well studied for automatic delineations of brain tumors in MRI scans. Through the datasets provided by the MICCAI BraTS challenges, among others, and with the rapid advancements in deep learning, different versions of the U-Net architecture^10^, such as nnU-Net^11^, Attention U-Net^12^, and Swin U-Netr^13^, have shown convincing results for medical image segmentation, like pre-operative glioma segmentation^11,13^. The BraTS challenge in 2023 provided a pre-operative glioma dataset with annotations of the enhancing tissue (ET), tumor core (TC), and whole tumor (WT), including the FLAIR hyperintensity volume. The winning team, using synthetic data augmentation, obtained a WT Dice score of 86.63 % with T1w, T1c, T2, and FLAIR scans as input^14^. In addition, the challenge provided a pre-operative meningioma dataset and pre-operative metastasis dataset with the same label types and input sequences. The winning WT lesion-wise Dice scores were 85.6 % and 60.2 %, respectively. However, all four sequence types are needed as input, and the models are tumor type-specific and for pre-operative scenarios only^15^. In the BraTS challenge 2024, post-operative glioma segmentation was included for the first time with annotations of enhancing tissue (ET), non-enhancing tumor core (NETC), surrounding non-enhancing FLAIR hyperintensity (SNFH), and resection cavity (RC). The best lesion-wise SNFH Dice score obtained was 87.6 % and the best lesion-wise WT score obtained was 87.0 % using the four MRI sequences as input^16^. Also here, all winning BraTS models have been tumor and acquisition time point specific models. However, there have been less or no focus on a unified model for FLAIR hyperintensity segmentation working on various brain tumor types and acquisition scan times. Some have created an automatic brain tumor segmentation in FLAIR images, but it is only for post-opertative glioblastomas^17^, or it is for 2D images, and they used only pre-preoperative gliomas^18^ or only 11 patients^19^. Others have created a segmentation model for white matter hyperintensity of vascular-origin or stroke origins in FLAIR scans, but not specifically for brain tumors^20,21^. Even though the pathological content of the FLAIR hyperintensity volume are different for various tumor types, a single unified model capable of segmenting FLAIR hyperintensity volumes across multiple tumor types both before and after treatments would be more clinically applicable than tumor type and acquisition time point specific models. This is particularly true for the FLAIR hyperintensity volume as the tissue diagnosis or the nature of the signals are often unknown. The FLAIR hyperintensity volume may also represent combinations of different processes that can be difficult to separate, like tumor and radiation gliosis for lower grade glioma, or gliosis or edema for meningioma^22^. Estimating accurately volumetric changes over time remains extremely clinically relevant, and having a single unified model working on all relevant acquisition time points and for various tumor types would be advantageous in both clinical practice and in clinical trails.

In this work, the aim was therefore to study automatic FLAIR hyperintensity segmentation, with the following main contributions: i) training of a unified model, ii) thorough validation of segmentation of both FH and SNFH structures, iii) performance investigation per tumor type and acquisition time point, iv) performance benchmarking against BraTS, and v) integrated and publicly available models in the open software Raidionics^23^.

## Materials and methods

This study was carried out in accordance with regional and national regulations. The participants involved provided their written informed consent to participate in this study. Approvals were obtained from the ethical committee of Western Sweden (Dnr: 702-18), the Norwegian regional ethics committee (REK ref. 2013/1348 and 2019/510), and from the American Institutional Review Board (IRB 2023P001681).

### Data

**Fig. 1:**
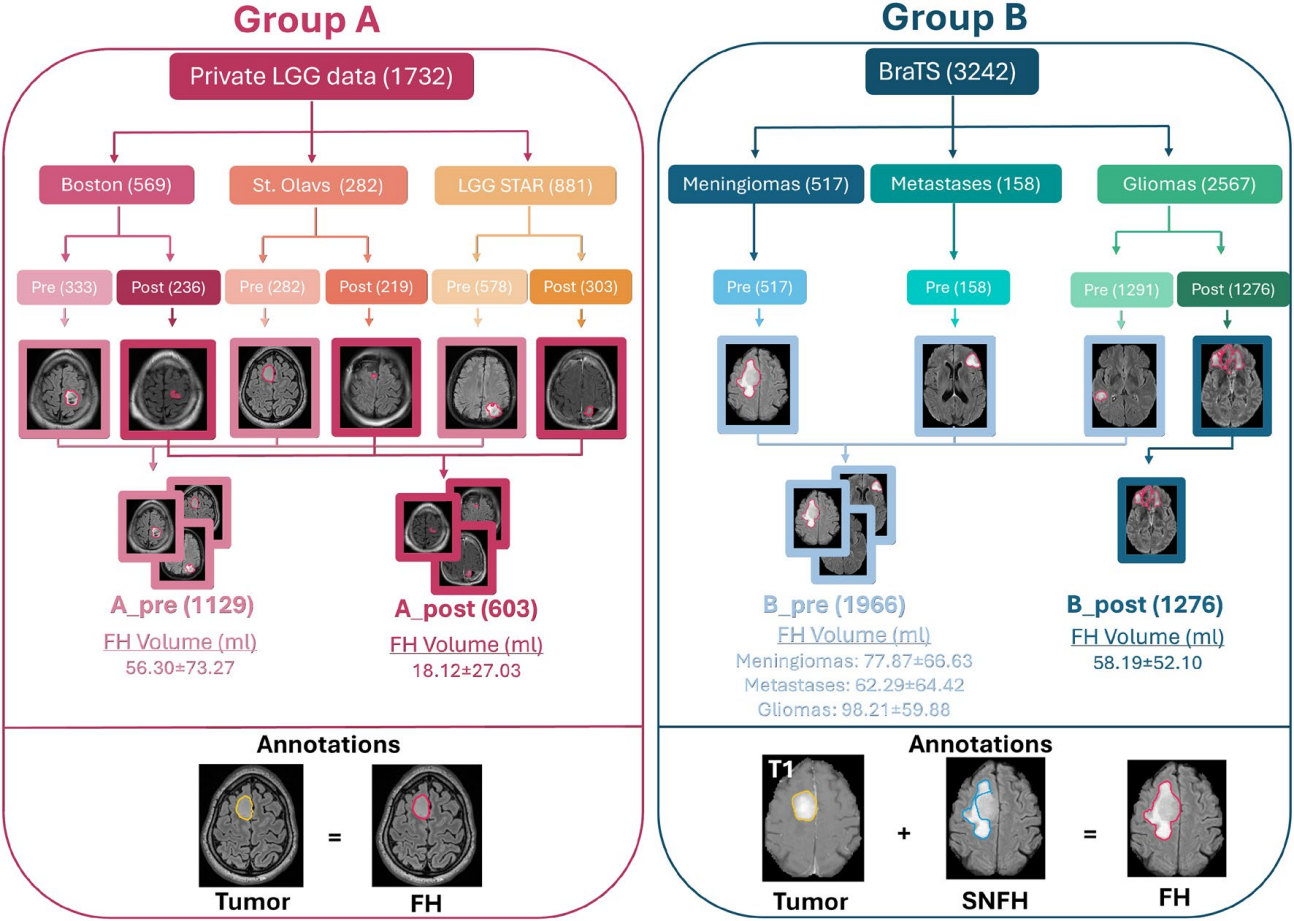
Illustration of the data used in this project showing the data origin with the number of images in parenthesis, the annotation types, distribution into different subgroups for model training, and average FLAIR hyperintensity volumes with standard deviation. Group A represents the private LGG dataset of tumors classified as supratentorial diffuse WHO grade II glioma according to the 2007 or 2016 WHO classification system^24,25^, with tumor annotations used as label for the FLAIR hyperintensity (FH) volume. Group B represents the BraTS data with annotations of the tumor and surrounding non-enhancing FLAIR hyperintensity (SNFH) volume, merged to produce the total FLAIR hyperintensity volume (FH). Group A has red-based colors, whereas group B has blue-based colors. Light colors represent pre-operative cases and dark colors represent post-operative cases.

Data from multiple sources with various tumor types, volumes, and acquisition time points were used. The data were divided into two groups, as presented in Figure 1. Group A comprised a private dataset of patients with supratentorial diffuse WHO grade II glioma classified according to the 2007 or 2016 WHO classification system^24,25^. The data originated from Brigham and Women’s Hospital (Boston, USA), St. Olavs hospital, Trondheim University Hospital (Trondheim, Norway), and from a Scandinavian multi-center cohort referred to as the LGG STAR group. It included annotated pre-operative and post-operative FLAIR scans, where 562 images were acquired up to 72 hours after surgery, 17 approximately one month after surgery, 19 approximately three months after surgery, and 5 approximately 6 months after surgery. The average dimensions of the FLAIR scans were $[365\times 381 \times 91]$ voxels, ranging between $[156;1024] \times [192;1024] \times [6;512]$ voxels, with an average spacing of $[0.70 \times 0.70 \times 4.04]$ mm^3^, ranging between $[0.24;1.30] \times [0.24;1.30] \times [0.50;18.00]$ mm^3^.

The datasets from group A contained annotations of tumor tissue which were used as label for the FLAIR hyperintensity volume, as seen in Figure 1. No standardized annotation protocol was defined for the datasets from group A other than the overarching objective of performing tumor tissue segmentation. Other MRI sequences were also used during the annotation process to aid the interpretation and reduce disambiguation. For post-operative cases, the pre-operative images were used to aid the decision process. The annotations were obtained from multiple expert annotators from multiple centers. Studies have shown that there is a large intra-rater variability in low-grade glioma segmentation with a median volume difference of 4.1 ml^7^, and large variations in the ground truths are expected.

Group B comprised data publicly available from the BraTS challenges in 2023 and 2024^26–31^, including annotated FLAIR scans of pre-operative meningiomas, metastases, and gliomas, and post-operative gliomas acquired at different time points after surgery, where 578 images were acquired up to 72 hours after surgery, 484 approximately 1 month after surgery, 109 approximately 3 months after surgery, and 70 approximately 6 months after surgery. The grade of the gliomas was not specified, but they were mostly contrast-enhancing. No patient overlap exists between group A and B. The average dimensions of the FLAIR scans were $[217 \times 231 \times 166]$ voxels, ranging between $[182;240] \times [218;240] \times [155;182]$ voxels, with a spacing of $[1.0\times 1.0 \times 1.0]$ mm^3^.

For group B, annotation of the tumor core (T) and surrounding non-enhancing FLAIR hyperintensity (SNFH), also called peritumoral edematous in BraTS 2023, were used. The tumor core comprises annotations of enhancing tissue and non-enhancing tumor core (called enhancing tumor and necrotic tissue in BraTS 2023). The tumor core annotations were obtained from hyperintensities in T1 and T1c images, while the SNFH annotations were obtained from the hyperintensities in the FLAIR scans around the tumor core, which could be edema, infiltrating tumor cells, and post-operative changes like ischemia^26–31^. These two annotations were merged to form a label of the entire FLAIR hyperintensity volume, as illustrated in Figure 1, which correspond to the whole tumor (WT) in BraTS.

To reduce annotation noise in the datasets, cases with a FH volume less than 0.1 ml were excluded, corresponding to annotations of less than 100 voxels. Cases with empty annotations (i.e., volume of 0 ml) were kept as true negative samples. In addition, a similar threshold of 0.1 ml was used over model predictions to determine positiveness (i.e., above threshold) or negativeness (i.e., below threshold) of the sample. All cases were positive, except for 81 cases from the post-operative images in group A.

### Methods

**Fig. 2:**
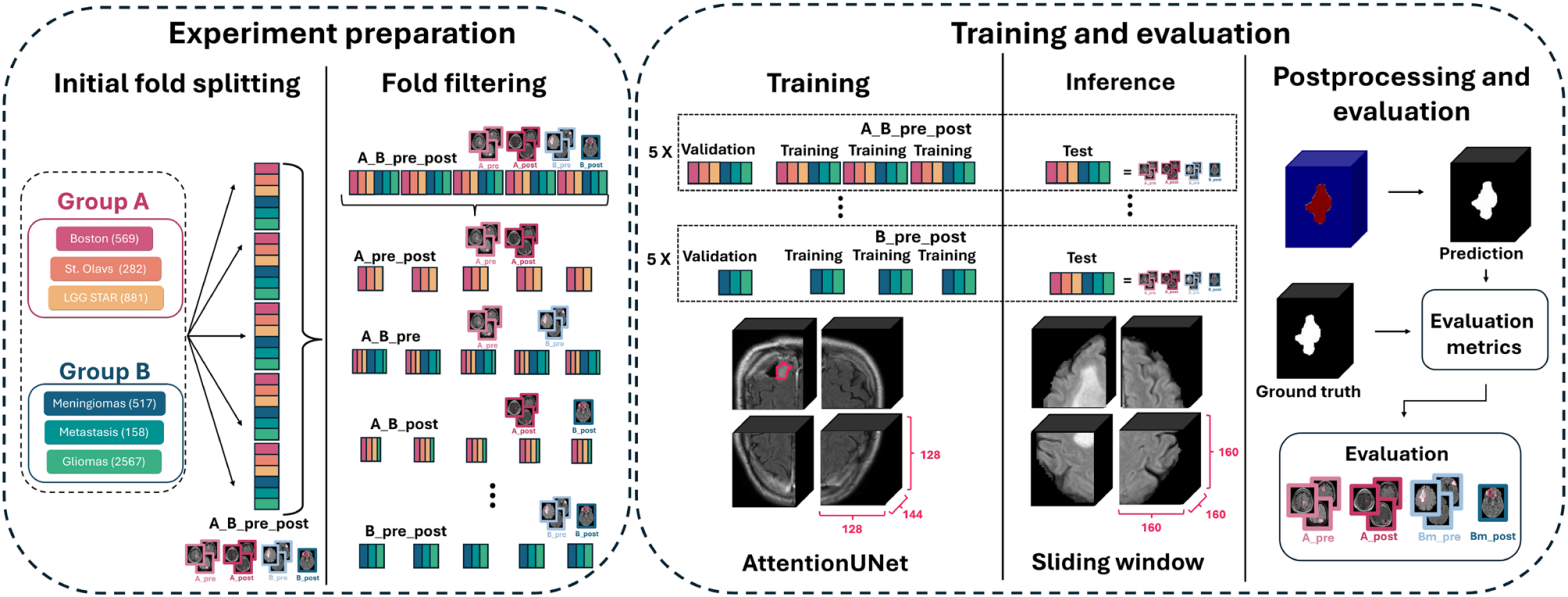
Illustration of the experiment preparation and the pipeline for training and evaluating segmentation models. An overview of the fold splitting strategy is presented. The datasets were evenly split with respect to tumor type, source, and size (represented by colors). Different subgroups of the data were used as input for training of the dataset-specific models. Note that each model was evaluated on all subgroups, and since the fold splits were kept fixed, the test sets are identical for all models. Only the training labels differed between the FH and SNFH segmentation models.

In this section, the experimental setup, preprocessing, model training, and evaluation are described. The pipeline for the data preparation and training of the segmentation models is illustrated in Figure 2.

#### Experimental setup and naming convention

In this study, different configurations of the datasets were used for training and evaluation of the models, as illustrated in Figure 2. All data were initially split into five evenly-distributed folds to perform three-way five-fold cross-validation. Five models were thus trained for the different configurations, where one fold was used as test set, one as validation set, and the remaining three as training set. This ensured that all data were used for training and all data were used as an independent test set. To ensure a fair comparison, the data were split patient-wise with an even distribution of the data source and tumor type in each fold, and balancing the number of smaller and larger tumors from each source in each fold. Multiple models were trained using different subgroups of the data, and the models were evaluated on all subgroups as shown in Figure 2. The same training strategy was used for all models, enforcing consistent fold splits across all experiments, ensuring comparability across the different configurations and ensuring an equal test set for all models.

Depending on the segmentation target, data with different label-types were used. To train FH segmentation models, the original tumor annotations from group A were directly used, while both SNFH and tumor annotation masks from group B were merged to form labels for the whole FLAIR hyperintensity volume, as illustrated in Figure 1. To train SNFH models, i.e., the surrounding FLAIR hyperintensity volume excluding contrast-enhancing tumor tissue, only group B was used with the original SNFH annotation masks.

Each model configuration was named descriptively by prepending the dataset group (e.g., A, B, or A_B for both) and appending the acquisition time point (e.g., pre, post, or pre_post for both). For further validation studies of specific tumor types, an additional prefix was prepended where Gli, Met, and Men represent glioma, metastasis, and meningioma, respectively. * was added to the model names for segmentation of SNFH.

#### Pre-processing

For pre-processing, all data were resampled to an isotropic voxel spacing of 1 mm^3^ with a first-order spline interpolation. To reduce background, tight cropping around the patient’s head was performed. In addition, intensity clipping within the range [0,99.5] % and zero-mean normalization of all nonzero values was performed.

#### Architecture design and training strategy

In this study, all models were trained with the Attention U-Net architecture^12^. The architecture was used with 5 levels with filter sizes of [16, 32, 64, 256, 512], instance normalization, a dropout rate of 0.2, and an input size of $128\times 128\times 144$ voxels. A combination of Dice and Cross-Entropy was used as loss function with the AdamW optimizer combined with an annealing scheduler and an initial learning rate of $5e{-4}$. The sigmoid function was used for the final activation layer, and the models were trained over 800 epochs with a batch size of 16 and a 2 step gradient accumulation strategy, giving an effective batch size of 32.

The models were trained with data augmentation. First, a random crop of $128\times 128\times 144$ voxels was applied to each input to match the network input size. Next, both geometric transformations and intensity-based transformations were applied. For geometric transformations, rotation within the range $[-20^{\circ }, 20^{\circ }]$, flipping along each axis, zoom scaling up to 15 %, and translation of up to 20 % of the axis dimension was applied, each with a 50 % probability. Intensity scaling and shifting up to 10 %, gaussian noise addition, gamma contrast adjustments in the range [0.5,2.0], and patch inversion or dropout with patch sizes of $10\times 10\times 10$ voxels and up to 75 elements, were applied with a 50 % probability for the intensity-based transformations.

#### Inference and postprocessing

For inference, the inference patch size was set to $160\times 160\times 160$ voxels, and a sliding-window approach with 50 % overlap between the patches was used.

As postprocessing, a binary brain mask was applied to the predictions, ensuring only the prediction probabilities inside the brain were kept. In addition, prediction probabilities with a volume lower than 0.05 ml^3^ or not visible in two consecutive 2D slices, were removed to reduce noise.

### Evaluation metrics

To compare and quantify model performance, different metrics were computed between the ground truth volume and a binary representation of the predicted probability maps obtained after inference with the trained models. The binary representation was computed for ten different probability thresholds between [0, 1]. The thresholds providing the highest scores were used to report the evaluation metric scores for all test samples for each fold.

To assess various aspects of performance, metrics focusing on classification capability, segmentation accuracy, and capturing clinically relevant outcomes were chosen.

**Classification:** Since the majority of cases were positive, except 81 cases in A_post, the evaluation focused solely on the models’ ability to correctly identify the target when present. Detection rate (also referred to as image-wise recall) was used, as the percentage of true positive cases (TP) among the total number of positive cases (P). A voxel-wise Dice score of $0.1\%$ between model prediction and ground truth was required for a positive case to be considered true positive.

**Segmentation:** To evaluate segmentation performance, when the model correctly detected the target, object-wise metrics were computed on the true positive samples. They describe the model’s ability to detect all components in case of multiple volumes of interest. A pairing strategy combined with a connected components approach was used to associate the ground truth and the model predictions’ components. From this, Dice score, recall, precision and 95th percentile Hausdorff distance (HD95), were computed and reported as mean ± standard deviation.

**Clinical use:** Regarding clinically-oriented metrics, reporting volume difference has potential for diagnosis and comparison with clinical guidelines. To evaluate this, the number of oversegmented and undersegmented cases and their respective volume differences were calculated among the true positive samples and reported as median [Interquartile Range].

## Experiments

The aim of this study was to develop and validate a unified FLAIR hyperintensity volume segmentation model that can be used for various brain tumor types, acquisition time points, and also for SNFH segmentation. In the following, the experiments performed are described.

### Evaluation of training set configurations

First, the unified model (A_B_pre_post) was trained using all subgroups of the data presented in Figure 1, thus A_pre, A_post, B_pre, and B_post, with FLAIR hyperintensity (FH) as label. To evaluate the performance of the unified model, dataset-specific models were trained using different configurations of the subgroups and compared to the unified model. All models were evaluated on all subgroups, thus A_pre, A_post, B_pre, and B_post. Since the fold splits were kept fixed, the test folds were the same for all models, ensuring a fair comparison, as shown in Figure 2. Only the segmentation metrics were used to compare the models.

The different configurations were selected based on a balance between training efficiency and the specific insights we aimed to obtain. Six dataset-specific models were trained with FLAIR hyperintensity (FH) as label using different combinations of A_pre, A_post, B_pre and B_post to obtain these models following the naming convention presented: A_pre, A_post, A_pre_post, A_B_pre, A_B_post, and B_pre_post.

In addition, for certain tumor types, segmenting only the surrounding non-enhancing FLAIR hyperintensity (SNFH) volume, without the tumor core, might be of interest. Another dataset-specific model was thus trained with SNFH as label using only B_pre and B_post since group A do not have SNFH labels, called B_pre_post*. To obtain this volume for the unified model, the tumor volumes were subtracted from the unified model’s predictions and evaluation metrics were computed against the original SNFH labels from group B. To ensure a fair comparison, the tumor volumes were also subtracted from the predictions of this dataset-specific model.

### Validation on various brain tumor types, sizes, and acquisition time points

Next, the unified model, trained on all data, was more thoroughly validated on different tumor types, targets, volume sizes, and for different pre- and post-operative scenarios using the classification, segmentation, and clinical metrics presented above.

### Benchmarking against BraTS

Lastly, the unified model was compared against the best scores from the BraTS Meningioma challenge 2023, BraTS Brain Metastasis challenge 2023, BraTS Adult Glioma challenge 2023, and BraTS Adult Glioma Post Treatment challenge 2024, thus ensuring proper benchmarking against current state-of-the-art models. Segmentation of the Whole Tumor (WT) from BraTS corresponds to the FLAIR hyperintensity volume (FH), and segmentation of the surrounding non-enhancing FLAIR hyperintensity volume (SNFH) is the same. The test sets used in the challenges are not publicly available, therefore the scores were collected from the official leaderboards available on Synapse showing the results on the validation sets^15,16^.

## Results

The training, inference, and validation processes were implemented in Pyhton 3.11 using PyTorch v2.4.0, PyTorch Lightning v2.4.0, and MONAI v1.4.0^32^. An Intel Core Processor (Broadwell, no TSX, IBRS) CPU with 16 cores, 64GB of RAM, Tesla A40 (46 GB) or A100 (80GB) dedicated GPUs, and NVMe hard-drives were used for the experiments.

### Evaluation of training set configurations

Segmentation performance for the various dataset configurations is summarized in Table 1. It shows that for both targets, the dataset-specific models achieved similar scores to the unified model (A_B_pre_post) on the test subgroups they were trained on. However, the dataset-specific models score clearly worse on other subgroups, while the unified model scores similar to the dataset-specific models for that subgroup. In addition, the table shows a variation in performance across the different test subgroups, varying from an average Dice score of $61.27\%$ for the A_post subgroup, to $89.51\%$ for the B_pre subgroup for the unified model.

**Table 1:** FLAIR hyperintensity (FH) volume and surrounding FLAIR hyperintensity (SNFH) volume segmentation performance summary for models trained with different configurations of the dataset. All models were evaluated on all the test subgroups. The unified model (A_B_pre_post), trained on all subgroups, is highlighted in bold. *represents the tumor volume subtraction for SNFH segmentation.

Model	Test subgroup	Target	Object-wise			
			Dice	Recall	Precision	HD95
A_pre	A_pre	FH	$84.52\pm 13.59$	$85.82\pm 15.32$	$86.28\pm 14.36$	$04.95\pm 05.42$
A_post	A_pre	FH	$75.31\pm 18.34$	$75.78\pm 23.53$	$81.77\pm 16.57$	$05.60\pm 07.09$
A_pre_post	A_pre	FH	$84.06\pm 13.55$	$85.26\pm 15.87$	$86.12\pm 14.27$	$05.28\pm 06.09$
A_B_pre	A_pre	FH	$84.90\pm 13.15$	$87.66\pm 14.14$	$85.17\pm 14.87$	$04.88\pm 05.71$
A_B_post	A_pre	FH	$74.92\pm 19.89$	$72.87\pm 24.49$	$84.60\pm 16.55$	$05.47\pm 07.23$
B_pre_post	A_pre	FH	$76.01\pm 20.16$	$85.76\pm 19.17$	$73.94\pm 23.38$	$08.46\pm 13.76$
**A_B_pre_post**	**A_pre**	**FH**	$\mathbf {84.47\pm 13.32}$	$\mathbf {88.79\pm 14.70}$	$\mathbf {83.48\pm 14.64}$	$\mathbf {04.97\pm 05.52}$
A_pre	B_pre	FH	$81.61\pm 18.53$	$78.93\pm 22.71$	$90.00\pm 13.13$	$07.69\pm 08.47$
A_post	B_pre	FH	$54.77\pm 29.21$	$46.24\pm 29.14$	$82.72\pm 30.81$	$15.77\pm 11.77$
A_pre_post	B_pre	FH	$74.93\pm 23.71$	$69.15\pm 26.79$	$89.75\pm 18.74$	$09.50\pm 09.57$
A_B_pre	B_pre	FH	$89.25\pm 11.48$	$87.80\pm 14.29$	$93.03\pm 08.52$	$03.91\pm 05.08$
A_B_post	B_pre	FH	$83.25\pm 15.29$	$83.65\pm 18.72$	$86.55\pm 13.02$	$06.45\pm 06.96$
B_pre_post	B_pre	FH	$89.26\pm 11.44$	$89.22\pm 13.68$	$91.47\pm 09.96$	$03.96\pm 05.03$
**A_B_pre_post**	**B_pre**	**FH**	$\mathbf {89.51\pm 10.60}$	$\mathbf {89.77\pm 12.99}$	$\mathbf {91.32\pm 09.26}$	$\mathbf {03.88\pm 04.70}$
A_pre	A_post	FH	$48.95\pm 27.31$	$78.55\pm 32.17$	$41.26\pm 27.13$	$12.76\pm 09.40$
A_post	A_post	FH	$61.37\pm 23.54$	$74.42\pm 22.73$	$61.15\pm 27.16$	$10.42\pm 08.42$
A_pre_post	A_post	FH	$62.19\pm 24.07$	$75.31\pm 23.71$	$62.19\pm 27.27$	$09.99\pm 08.25$
A_B_pre	A_post	FH	$51.59\pm 27.76$	$80.51\pm 29.27$	$43.73\pm 28.32$	$07.79\pm 07.90$
A_B_post	A_post	FH	$61.26\pm 23.96$	$73.02\pm 24.79$	$61.69\pm 27.00$	$10.15\pm 07.98$
B_pre_post	A_post	FH	$39.43\pm 29.13$	$67.51\pm 38.78$	$33.68\pm 29.21$	$12.75\pm 12.47$
**A_B_pre_post**	**A_post**	**FH**	$\mathbf {61.27\pm 24.35}$	$\mathbf {78.26\pm 24.55}$	$\mathbf {58.60\pm 26.63}$	$\mathbf {10.26\pm 08.44}$
A_pre	B_post	FH	$64.15\pm 24.92$	$57.24\pm 27.59$	$85.90\pm 21.56$	$17.27\pm 14.63$
A_post	B_post	FH	$58.82\pm 24.53$	$50.40\pm 26.26$	$85.54\pm 23.07$	$18.26\pm 13.54$
A_pre_post	B_post	FH	$66.13\pm 22.42$	$57.89\pm 24.94$	$89.38\pm 17.23$	$15.93\pm 13.89$
A_B_pre	B_post	FH	$73.28\pm 20.23$	$68.13\pm 22.96$	$88.10\pm 17.58$	$10.87\pm 11.48$
A_B_post	B_post	FH	$84.41\pm 12.02$	$85.27\pm 12.88$	$86.57\pm 13.06$	$04.77\pm 06.23$
B_pre_post	B_post	FH	$84.75\pm 12.14$	$86.35\pm 12.65$	$86.21\pm 13.35$	$04.76\pm 06.10$
**A_B_pre_post**	**B_post**	**FH**	$\mathbf {84.60\pm 11.78}$	$\mathbf {86.55\pm 12.42}$	$\mathbf {85.62\pm 13.19}$	$\mathbf {04.86\pm 06.47}$
B_pre_post*	B_pre	SNFH	$81.02\pm 17.75$	$81.96\pm 18.59$	$85.08\pm 17.26$	$05.43\pm 06.66$
A_B_pre_post*	**B_pre**	**SNFH**	$\mathbf {83.40\pm 16.87}$	$\mathbf {86.96\pm 16.06}$	$\mathbf {84.98\pm 17.47}$	$\mathbf {05.20\pm 07.05}$
B_pre_post*	B_post	SNFH	$83.23\pm 13.19$	$85.05\pm 14.55$	$85.36\pm 13.21$	$05.06\pm 06.83$
A_B_pre_post*	**B_post**	**SNFH**	$\mathbf {83.94\pm 12.76}$	$\mathbf {85.43\pm 13.44}$	$\mathbf {85.96\pm 13.76}$	$\mathbf {04.95\pm 06.87}$

### Validation on various brain tumor types, sizes, and acquisition time points

**Fig. 3:**
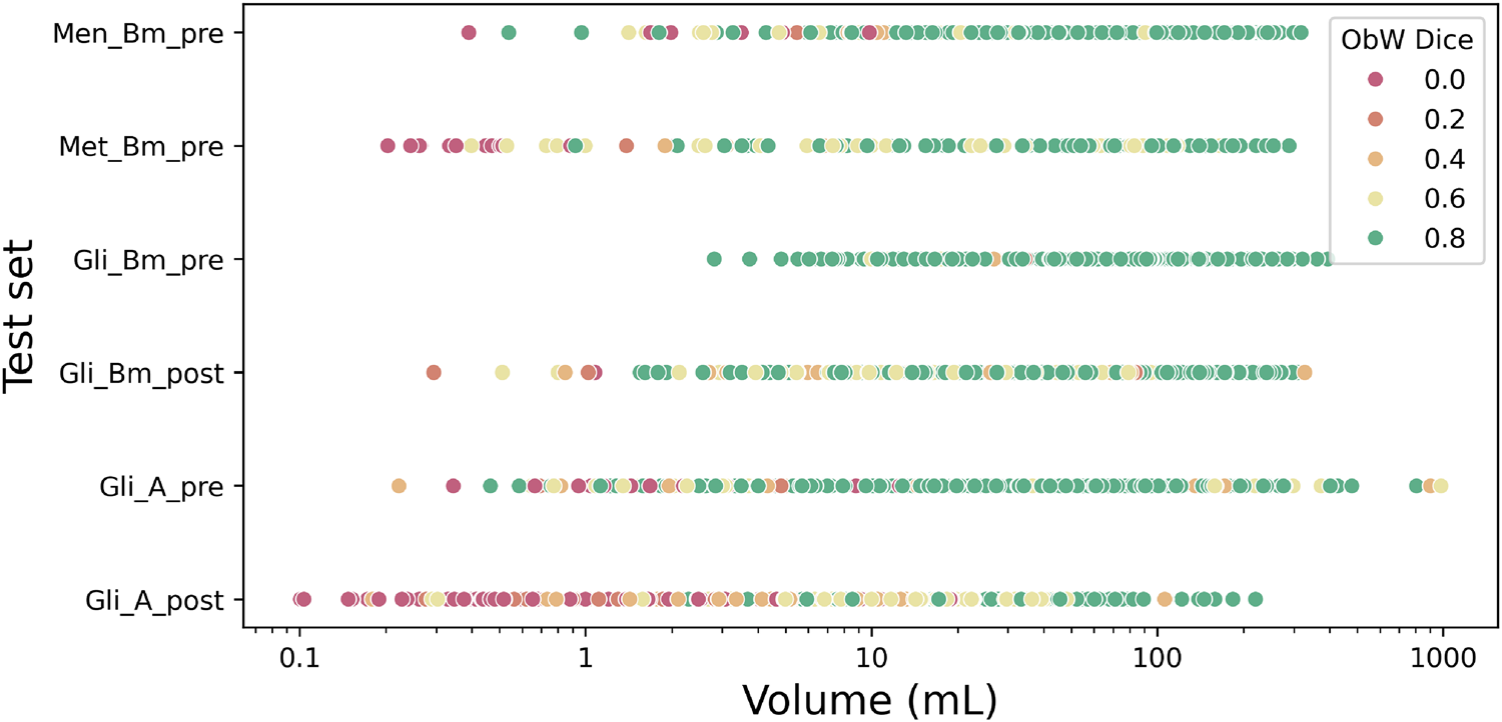
Scatterplot showing the object-wice Dice scores for the unified model (A_B_pre_post) on different test sets grouped by brain tumor type pre- and post-operatively along the Y-axis and different volumes with a logarithmic scale along the X-axis. All test cases are shown, including false negative samples.

The performance of the unified model (A_B_pre_post) on different tumor types, acquisition time points, and with FH or SNFH as target are summarized in Table 2. It shows a difference in performance across the groups. For both targets, the unified model scored best on Gli_B_pre, obtaining the best average object-wise Dice scores with high detection rates, high and similar recall and precision scores, and low HD95 scores. The number of over- and undersegmented cases are equally distributed, with small volume differences compared to the average volume. Moreover, the unified model achieved strong scores on Men_B_pre and Gli_B_post and slightly worse on Met_B_pre with a lower detection rate, for both FH and SNFH. For FH segmentation of dataset A, the unified model achieved good scores for Gli_A_pre, but struggled with Gli_A_post where it obtained the lowest and most variable scores, with a high recall, but low precision, and obtained a high number of oversegmented cases compared to the undersegmented cases. In addition, the unified model generally scored lower for SNFH than for FH. Moreover, Figure 3 shows the relationship between Dice score and volume distributions for the different test sets comprising of different tumor types and acquisition time points with FH as target. It shows that cases with smaller volumes generally give lower Dice scores.

**Table 2:** Segmentation performance with the FLAIR hyperintensity (FH) and the surrounding non-enhancing FLAIR hyperintensity (SNFH) volumes as target for the unified (A_B_pre_post) model, on meningiomas (Men), metastases (Met), and gliomas (Gli) from dataset A and B, and for both pre- and post-operative acquisition time points. Detection rate is calculated on the positive cases, and the other metrics are calculated on only true positive cases.

Test set	Target	Detection rate	Dice	Recall	Precision	HD95	Oversegmentation		Undersegmentation	
							$\Delta$ (ml)	# Samples	$\Delta$ (ml)	# Samples
Men_B_pre	FH	98.07	$88.65\pm 12.47$	$89.00\pm 15.38$	$90.52\pm 09.26$	$04.05\pm 04.75$	3.30 [1.34–6.89]	272	3.24 [1.39–8.04]	235
Met_B_pre	FH	92.41	$80.08\pm 13.75$	$80.81\pm 16.90$	$84.73\pm 13.61$	$04.33\pm 03.94$	4.11 [1.01–10.25]	71	4.87 [1.45–10.64]	75
Gli_B_pre	FH	99.85	$90.92\pm 08.56$	$91.09\pm 10.79$	$92.39\pm 08.21$	$03.76\pm 04.84$	3.74 [1.33–7.35]	569	5.16 [2.02–10.34]	720
Gli_B_post	FH	99.84	$84.60\pm 11.78$	$86.55\pm 12.42$	$85.62\pm 13.19$	$04.86\pm 06.47$	3.21 [1.20–6.96]	624	3.01 [1.34–6.18]	650
Gli_A_pre	FH	98.05	$84.47\pm 13.32$	$88.79\pm 14.70$	$83.48\pm 14.64$	$04.97\pm 05.52$	3.14 [1.23–8.28]	605	3.26 [1.10–7.80]	502
Gli_A_post	FH	90.61	$61.27\pm 24.35$	$78.26\pm 24.55$	$58.60\pm 26.63$	$10.26\pm 08.44$	3.97 [1.43–9.60]	331	2.39 [1.00–4.56]	142
Men_B_pre	SNFH	96.91	$78.96\pm 23.13$	$90.83\pm 16.73$	$76.20\pm 24.33$	$08.05\pm 09.61$	3.42 [1.48–7.51]	409	1.12 [0.39–2.95]	92
Met_B_pre	SNFH	92.41	$75.57\pm 19.28$	$77.77\pm 20.87$	$80.22\pm 19.31$	$04.24\pm 03.78$	3.18 [1.07–9.34]	79	3.13 [1.06–9.42]	67
Gli_B_pre	SNFH	99.85	$86.02\pm 12.37$	$86.50\pm 14.54$	$88.94\pm 11.62$	$04.24\pm 05.75$	3.39 [1.31–7.00]	617	4.09 [1.61–8.94]	672
Gli_B_post	SNFH	99.84	$83.94\pm 12.76$	$85.43\pm 13.44$	$85.96\pm 13.76$	$04.95\pm 06.87$	3.24 [1.24–7.21]	664	2.29 [1.05–4.84]	610

**Fig. 4:**
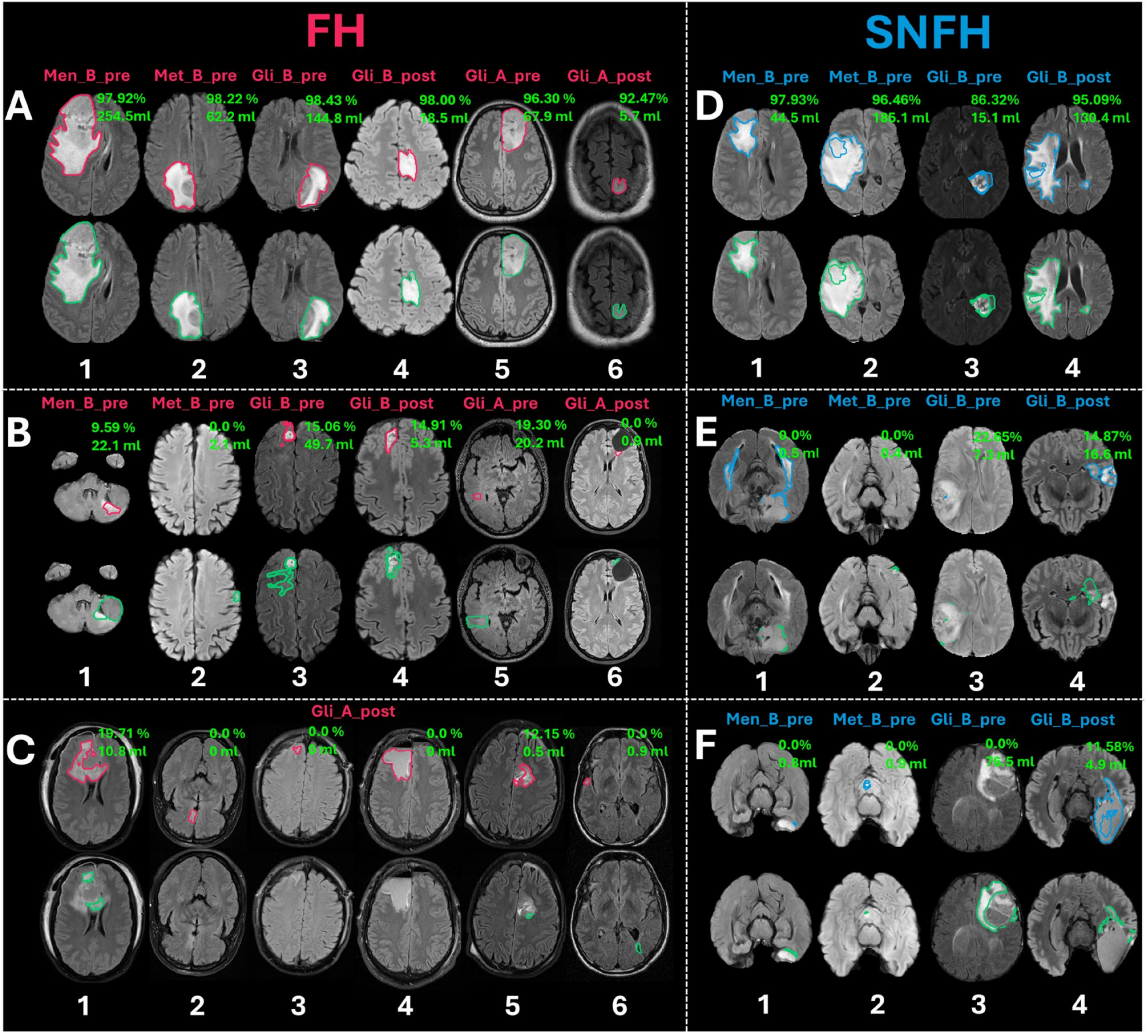
Examples of predictions of FH in pink and SNFH in blue, and the ground truth in green. A1-A6 show examples with high Dice scores, and B1-B6 show examples with low Dice scores for the different tumor types and acquisition time points for FH segmentation. C1-C6 show more examples with low Dice scores for FH segmentation from Gli_A_post. D1-D4 show examples with high Dice scores. Finally, E1-E4 and F1-F4 show examples with low Dice scores for SNFH segmentation for the different tumor types and acquisition time points.

Some examples of predictions of FH and SNFH can be seen in Figure 4. It shows examples with high and low Dice scores for all tumor types and acquisition time points, and some extra examples of the Gli_A_post cases. Some repeating patterns were observed for the cases with poor predictions. For some, the ground truth masks are debatable (B1, B3, B5, C1, E4). Others, typically metastasis with multiple very small lesions or post-operative cases with very small and multiple residual tumors, are missing some lesions in either the ground truth masks, the predictions or both (B2, B6, C6, E2, F2). For SNFH segmentation, some have a very small remaining mask after tumor core substraction (E1, E3). Also, other cases that are very small illustrates that small differences in the overlap gives a large decrease in Dice score (C5, F1, F2). In others, the surgical cavity with hyperintense FLAIR-signal as seen early postoperatively, is included in the automatic segmentation masks (B4, C3, C4, C5, F4). Segmentation of other pre-existing ischemic white matter changes are also observed (C2, E1). The model also struggles when there are some artifacts in the image (F3).

Table 3 shows the performance on FH segmentation on scans acquired before (Pre), early after (Early post), and later after (Late post) surgery, which includes the scans acquired around 1 to 6 months after surgery (Post1, Post3, and Post 6). Among the 81 negative cases of the post-operative gliomas from group A, 55 were considered as false positive from Early post, 1 from Post 1 and 1 from Post 6. Some examples of false positive and false negative cases, can be seen in Figure 4 (B2, C2, C3, C4, E2, F3).

**Table 3:** Segmentation performance for the unified model on dataset A, B and the entire dataset grouped by different acquisition time points, where “Pre” cases were acquired pre-operatively, “Early post” cases were acquired right after surgery, and “Late post” cases were acquired around 1–6 months after surgery. For the entire dataset “Late post” is also split in “Post 1”,“Post 3” and “Post 6”, which indicate the rounded-up number of months post-surgery at which the MRI scan is taken. Note that meningiomas and metastases do not have post-operative images and are thus only in the pre-operative group.

Acquisition time point	Detection rate	Dice	Recall	Precision	HD95	Oversegmentation		Undersegmentation	
						$\Delta$(ml)	# Samples	$\Delta$(ml)	# Samples
**Dataset A**									
Pre	98.05	$\mathbf {84.47\pm 13.32}$	$\mathbf {88.79\pm 14.70}$	$\mathbf {83.48\pm 14.64}$	$\mathbf {04.97\pm 05.52}$	3.14 [1.23–8.28]	605	3.26 [1.10–7.80]	502
Early post	90.72	$60.60\pm 24.54$	$77.96\pm 24.75$	$57.99\pm 26.85$	$10.39\pm 08.46$	3.76 [1.32–9.42]	310	2.39 [1.02–4.56]	130
Late post	89.19	$70.38\pm 21.13$	$80.33\pm 23.04$	$70.94\pm 22.88$	$08.53\pm 08.09$	5.87 [4.28–12.44]	21	2.52 [0.78–4.37]	12
**Dataset B**									
Pre	98.78	$\mathbf {89.51\pm 10.60}$	$\mathbf {89.77\pm 12.99}$	$\mathbf {91.32\pm 09.26}$	$\mathbf {03.88\pm 04.70}$	3.58 [1.33–7.32]	912	4.64 [1.75–10.03]	1030
Early post	99.84	$83.59\pm 12.92$	$86.17\pm 12.97$	$83.90\pm 14.76$	$05.01\pm 06.45$	3.36 [1.35–7.28]	316	2.99 [1.39–5.57]	296
Late post	99.85	$85.60\pm 10.70$	$85.9\pm 12.18$	$88.3\pm 11.2$	$04.73\pm 06.49$	3.01 [1.07–6.80]	308	3.13 [1.20–6.44]	354
**Dataset A and B**									
Pre	98.51	$87.68\pm 11.92$	$89.42\pm 13.65$	$88.47\pm 12.12$	$04.27\pm 05.08$	3.41 [1.30–7.63]	1517	4.21 [1.50–9.46]	1532
Early post	95.81	$73.98\pm 21.86$	$82.72\pm 19.28$	$73.07\pm 24.34$	$07.20\pm 07.81$	3.57 [1.33–7.78]	626	2.77 [1.24–5.45]	426
Late post	99.29	$84.88\pm 11.83$	$85.64\pm 12.93$	$87.48\pm 12.54$	$04.91\pm 06.54$	3.21 [1.09–7.41]	329	3.06 [1.20–6.37]	366
Post 1	99.80	$84.20\pm 11.53$	$86.08\pm 12.33$	$85.78\pm 12.65$	$04.91\pm 06.34$	3.22 [1.07–6.81]	242	3.17 [1.19–6.32]	257
Post 3	97.62	$85.76\pm 11.37$	$88.13\pm 12.58$	$86.47\pm 12.88$	$05.38\pm 07.70$	3.39 [1.24–8.02]	55	2.73 [1.41–6.73]	68
Post 6	98.64	$87.27\pm 11.43$	$88.84\pm 11.54$	$88.46\pm 13.23$	$04.12\pm 05.76$	2.09 [1.24–7.07]	32	2.23 [1.03–6.22]	41

### Benchmarking against BraTS

The performance of our unified model on our BraTS test sets compared to the best scores on the validation sets from the BraTS challenges are presented in Table 4. It shows that our model obtained comparable results for FH segmentation of pre-operative gliomas, meningiomas and metastases, and for FH and SNFH segmentation of post-operative gliomas, compared to the validation set scores from the BraTS challenge.

**Table 4:** Our unified model (A_B_pre_post) compared to the results on the validation set for the BraTS challenges 2023 and 2024. Whole tumor (WT) corresponds to the FLAIR hyperintensity (FH) volume. *Represent the tumor subtraction from the predictions to get the SNFH volume.

Model	Tumor type	Target	# Samples	# Inputs	Test/Val	Object/lesion-wise
						Dice
Our	Meningioma	FH/WT	517	1	Test	$88.65\pm 12.47$
BraTS 2023	Meningioma	FH/WT	141	4	Val	85.6
Our	Metastasis	FH/WT	158	1	Test	$80.08\pm 13.75$
BraTS 2023	Metastasis	FH/WT	31	4	Val	60.2
Our	Glioma pre	FH/WT	1291	1	Test	$90.92\pm 08.56$
BraTS 2023	Glioma pre	FH/WT	219	4	Val	91
Our	Glioma post	FH/WT	1276	1	Test	$84.57\pm 11.92$
BraTS 2024	Glioma post	FH/WT	188	4	Val	87.6
Our*	Glioma post	SNFH	1276	1	Test	$84.60\pm 11.78$
BraTS 2024	Glioma post	SNFH	188	4	Val	87.2

## Discussion

This study aimed to develop and validate a robust unified FLAIR hyperintensity segmentation model for various brain tumors and acquisition time points, that also can be used for SNFH segmentation. First, dataset-specific models were trained and compared against the unified model, and the model was used with tumor subtraction to evaluate the performance on the surrounding non-enhancing FLAIR hyperintensity volume. Next, the unified model was validated on different tumor types and acquisition time points. Finally, the model was benchmarked against the top performing models trained on the BraTS dataset.

The datasets used in this study comprised a large and diverse patient population, covering all major CNS tumor types, including meningiomas, metastases, and various WHO grades of gliomas. However, the data used originates from different sources without a common annotation protocol for the FLAIR hyperintensity volume. For the BraTS annotations in group B, the original labels were of the SNFH and the tumor volume which were merged to get the whole FLAIR hyperintensity volume. This could result in differences in the ground truth mask compared with those that would be obtained if the FLAIR hyperintensity volumes were annotated directly. For Group A, comprising low grade gliomas, the original labels were of the tumor volume that were used for the whole FLAIR hyperintensity volume. For low grade gliomas, the entire pre-operative FLAIR hyperintensity volume is associated with the whole tumor volume because these tumors have no or little edema. Nevertheless, some edema can occur during treatment, and the post-operative tumor volume could thus be smaller than the early post-operative FLAIR hyperintensity volume^33^. In addition, the lack of a standardized annotation protocol in such a multi-centric dataset surely introduced noise and unwanted variations in the annotations. For example, in case of multifocal tumors, all foci or just the one planned for resection could be annotated, as seen in Figure 4. Also, there could be variations if the entire tumor volume was annotated or just the volume targeted for resection. Such inconsistencies, possibly underestimating ground truth volumes, could thus induce noise in the training and is not ideal for evaluation, where the performance may be better than what the scores indicate, like the case for C1 and C2 in Figure 4. Re-annotating all data with the same annotation protocol is time consuming and requires expert-knowledge and was therefore not achievable within this study. In addition, as seen, there is a high intra-rater variability for FLAIR hyperintensity segmentation with a median volume difference of 4.1 ml^7^, and a complete overlap in predictions and ground truth volumes are thus not realistic.

The unified model (A_B_pre_post) achieved comparable segmentation performance to the dataset-specific models on their respective datasets, with both FH and SNFH as target, as shown in Table 1. However, as seen, the dataset-specific models obtained clearly worse scores than the unified model on other test subsets. Even though some of the dataset-specific models offer minor improvements in performance on their respective dataset, the unified model aligns good segmentation performance with versatility enabling generalization across tumor types and acquisition time points. Although the cellular content of the pathological FLAIR signal may often be different in glioblastoma, lower grade glioma, and other brain tumor types, the FLAIR images only show either a non-elevated signal compared to normal brain or a hyperintense signal. The interpretation of what the FLAIR signal is can be different depending on the setting (e.g. pre-/post-operative) or the expected pathology. For proper disambiguation of the FLAIR signal, radiologists need concomitant access to more sequences and setting knowledge. Still, estimating tissue diagnoses from radiology alone is not always straight forward, for example in non-enhancing glioblastomas. Separating non-enhancing tumor from edema in glioblastoma is often very difficult and probably not very reproducible. This is why definitions of flairectomies in glioblastomas by RANO^3^, focus on the FLAIR volume, not its potential contents. A simple assessment of the FLAIR hyperintensity volume across different types of settings and pathologies is therefore more useful. In addition, this simplifies deployment in a clinical setting, which is especially beneficial in a diagnostic setting when the tumor type is still unknown. A unified model is thus more desirable for this application.

The validation of the unified model on different tumor types, pre- and post-operative showed generally good scores with both FH and SNFH as targets, however there were some differences across the groups. Figure 3 indicates that the Dice score is largely affected by the volume. Across all groups, smaller volumes generally corresponded to lower Dice scores, while larger volumes tended to yield higher scores. It seems that for all groups, cases with a volume lower than around 1 ml are challenging to segment. This was also seen in the examples in Figure 4. In addition, the lower performing groups had overall smaller volumes than the higher performing groups. From Figure 1, it can be seen that there is a large difference in the average FLAIR hyperintensity volume of the different tumor types and acquisition time points, where the post-operative gliomas from group A have the lowest average volume of $18.12\pm 27.03$ ml, and the pre-operative gliomas from group B have the largest average volume of $98.12\pm 59.88$ ml. This could explain why the pre-operative gliomas from group B obtained the highest Dice scores and why the post-operative gliomas from group A obtained the worse scores. In addition, it can be seen that the unified model fails to segment most of the cases under 1 ml for the meningiomas and post-operative gliomas from group B, which could explain the lower detection rate for these groups. Moreover, it can explain why the SNFH scores are generally lower than the FH scores, because the volumes are generally lower. This is a well-known phenomenon in machine learning since smaller objects tend to disappear before the final, most abstract, feature maps. In addition, small objects give a high class imbalance which is a challenge in the learning process. Also, Dice score is highly sensitive to errors in small structures, and Dice loss is suboptimal as it penalizes more heavily very small segmentation differences^34^. On the other side, large structures could obtain a high Dice score, even with large differences. It is thus important to not only evaluate segmentation performance using Dice score, and it is why we also included other metrics. Nevertheless, this indicates that the volume affects the Dice scores more than the tumor type and acquisition time points. In addition, group A has a higher average slice thickness which affects the quality of the images and might affect the performance of the model and the calculation of the evaluation metrics. This could also explain why the results are generally better for the data from group B.

However, FH appears to be particularly difficult to segment in dataset Gli_A_post, as performance is generally lower than for the other datasets, with similar structure volumes. Several factors can cause such performance drop, starting by the tendency of the unified model to perform oversegmentation in this subgroup, as seen in Table 2. According to Figure 4, the model tends, in some cases, to produce a segmentation when the ground truth is empty, to segment a larger volume than the ground truth, or to segment another structure than the ground truth. However, some clearly visible FH regions were not included in the ground truth, which can be ascribed to the lack of standardized annotation protocol as previously discussed. The performance on this specific subgroup could thus be better than reflected in the reported metrics. Furthermore, the unified model’s exposure to datasets with labels approximating better the true FH volume could be an advantage for post-operative non-contrast-enhancing glioma segmentation, enabling the model to segment FH regions more accurately. In addition, the inter-rater variability of manual FH segmentation can be substantial^7^, with some cases with a debatable ground truth. The results also shows that Gli_A_post, has an over- and undersegmentation with a median volume differences of 3.97 ml and 2.39 ml, respectively. This is comparable to the measured intra-rater variability with a volume difference of 4.1 ml^7^. For segmentation of diffuse FLAIR hyperintensity volumes, high precision (i.e. reproducibility) may be at least as important as high accuracy to increase reproducibility of image assessments in clinical practice, clinical trials, and in treatment registries.

Post-operative scans are particularly difficult to segment as the edges are often unclear and disturbed by swelling, ischemia, blood products, treatment induced proteins, hemostatic agents, and other cavity-fluids that could increase FLAIR signals. This is supported by the results from Table 3, indicating higher scores over pre-operative scans than post-operative, for both groups. Post-operatively, early scans taken up to 72 hours after surgery obtained the lowest scores, while performance improves for late post-operative scans, acquired 1 to 6 months after surgery. An explanation can be that the disturbing factors that occur right after treatment have had time to settle and are less pronounced in later scans, giving clearer edges and an easier segmentation task. This can also be seen in Figure 4, which shows that it is more difficult to identify blood products, cavity fluid, or FLAIR hyperintensity caused by edema or tumor tissue in early post-operative scans. The model also wrongly segments the cavity fluid in some of the examples, which can be explained by that proteins in surgical cavities increase FLAIR-signals.

Compared to BraTS, Table 4 shows that our model obtained similar scores on all tasks, and higher scores over the metastasis category. However, the BraTS scores might be slightly higher from being computing over the validation set and not the test set, in contrast to our results that were derived from a test set never seen during training. The validation sets are also smaller than our test sets, which makes them more sensitive to random fluctuations, noise, and the influence of individual cases. Furthermore, the BraTS models are given more information with four MRI sequences as input(i.e., T1w, T1c, T2, and FLAIR), whereas our model uses only FLAIR scans. In addition, the implementation of the pairing strategy to compute the object-wise scores in our work and lesion-wise scores in BraTS may slightly differ, which can impact the results. For instance, thresholds used to determine overlapping structures will affect the final scores. This could explain why our unified model obtained an object-wise Dice score of 80.08% on the metastasis subgroup where multifocal tumors are common, compared to 60.2% for BraTS. Differences could also stem from our choice to report performance over true positives only, whereby the detection rate was lower than for the other groups. The thresholds used for assessing positiveness or negativeness will also influence the scores. Our results are thus not directly comparable to BraTS, nevertheless such benchmarking still provides a useful indication of our model’s performance, and shows that our models obtain good scores on the different BraTS tasks.

Regarding clinical usage, the unified model has achieved promising results and could be useful in a clinical context to assess the FLAIR hyperintensity volume, both in pre-operative and post-operative scenarios, and holds potential to quantify changes over time. The median volume differences are low compared to the average FH volumes, supporting the use of the segmented volumes for assessing clinical guidelines. For non-contrast-enhancing tumors, such as low grade gliomas, this volume is important to assess the tumor volume^3^. However, a limitation in this study is that some clinicians prefer to use T2 scans instead of FLAIR scans to assess the tumor volume. For this study, more FLAIR images were available than T2 scans, and only FLAIR scans were thus used. For future work, it would be interesting to validate our models against annotations in T2 scans directly and compare the results to annotations in FLAIR scans. Moreover, for contrast-enhancing tumors (such as meningioma, glioblastoma and metastasis), it could be interesting to only analyze the surrounding FLAIR hyperintensity volume. Since the contrast-enhancing tumor volumes often are known, either from manual annotations or from high-performing segmentation software, such as Raidionics^23^, the tumor volume can be subtracted from the predictions from the unified model to obtain this volume. As seen, the unified model performs well, and it also achieved better results than the dataset-specific model trained using only the SNFH labels presented in Figure 1. The surrounding FLAIR hyperintensity volume is also important after surgery for the contrast-enhancing tumors, for example to assess edema which is linked to survival in glioblastomas^3,4^. In addition, across tumors, radiation induced leukoenceohalopathy seen as progressing peritumoral FLAIR hyperintensities is linked to neurocognitice function^35^. However, more thorough clinical validation is needed before clinical implementation^36,37^. An important part of the clinical validation would be to define what the FLAIR hyperintensity volume is. There are biological differences depending on the tumor type, and there are also differences depending on the center, neurosurgeon, and if the goal is to get the removable FLAIR hyperintensity volume, or the entire volume, especially for the non- or less enhancing WHO grade 2–3 gliomas, which must be considered before a more thorough clinical validation.

Even though the unified models achieved good performance, there is room for further improvements, especially for cases with a volume smaller than around 1 ml. First, as discussed, the main limitation in this study is that the data used originates from different sources without a common annotation protocol for the FLAIR hyperintensity volume. As seen, this resulted in noise in the dataset, which was typically visible for Gli_A_post. For future work, new labels can be acquired with a standardized annotation protocol to reduce the noise in the annotations. This can be obtained more rapidly by manually adjusting predictions from the unified model, or by interactive active learning. Also, the dataset should be expanded to obtain more cases without any FLAIR hyperintensities to balance the dataset. Images from true negatives could be used since there are often some remaining FLAIR hyperintensity volumes in the post-operative images, especially for contrast-enhancing tumors which often still have edema after surgery. Other training strategies more optimal for small structures can also be tested, such as multi-scale approaches, identifying big structures first and then at a lower resolution refining the first mask, different loss functions or just focusing on small objects. In addition, the model can be improved further by providing multiple MRI sequences as inputs and extending to multi-class segmentation of the FLAIR hyperintensity volume, the contrast-enhancing volume and cavity. This could help for the cases where the cavity is segmented. If some MRI sequences are missing, synthetic MRI sequences can be generated using denoising diffusion models. In addition, denoising diffusion models could be used to translate 2.5D MR scans, with high slice thickness, into 3D scans with appropriate resolution, avoiding the added blurriness from naive resampling techniques.

## Conclusion

This research showed that our unified FLAIR hyperintensity segmentation model aligns good segmentation performance across different brain tumor types and acquisition time points with good generalization. The unified model simplifies deployment in a clinical setting and has potential for clinical use. The unified model is integrated and publicly available in the open software Raidionics^23^.

## Data Availability

The data analyzed in this study is subject to the following licenses/restrictions: patient data are protected under GDPR and cannot be publicly distributed. Requests to access these datasets should be directed to David Bouget (david.bouget@sintef.no) for consideration. The BraTS Challenge datasets are available at the following URL: https://www.synapse.org/Synapse:syn53708126/wiki/626320. The Raidionics environment with all related information is available at https://github.com/raidionics. More specifically, all trained models can be accessed at https://github.com/raidionics/Raidionics-models/releases/tag/v1.3.0-rc , the Raidionics software can be found at https://github.com/raidionics/Raidionics . Finally, the source code used to compute the validation metrics is available at https://github.com/dbouget/validation_metrics_computation.
